# Prevalence of subclinical pulmonary tuberculosis and its association with HIV in household contacts of index tuberculosis patients in two South African provinces: a secondary, cross-sectional analysis of a cluster-randomised trial

**DOI:** 10.1186/s44263-023-00022-5

**Published:** 2023-11-01

**Authors:** Naomi Carter, Emily L. Webb, Limakatso Lebina, Kegaugetswe Motsomi, Zama Bosch, Neil A. Martinson, Peter MacPherson

**Affiliations:** 1 Liverpool School of Tropical Medicine, Liverpool, UK; 2 MRC International Statistics and Epidemiology Group, London School of Hygiene and Tropical Medicine, London, UK; 3 Clinical Trials Unit, Africa Health Research Institute, Johannesburg, South Africa; 4 Perinatal HIV Research Unit (PHRU), University of the Witwatersrand, Johannesburg, South Africa; 5 Centre for the AIDS Programme of Research in South Africa (CAPRISA), Doris Duke Medical Research Institute, Nelson R Mandela School of Medicine, University of KwaZulu-Natal, Durban, South Africa; 6 Johns Hopkins University Center for TB Research, Baltimore, MD USA; 7 School of Health & Wellbeing, University of Glasgow, Glasgow, UK; 8 Clinical Research Department, London School of Hygiene and Tropical Medicine, London, UK

**Keywords:** Tuberculosis, Subclinical, HIV

## Abstract

**Background:**

People with subclinical tuberculosis (TB) have microbiological evidence of disease caused by *Mycobacterium tuberculosis*, but either do not have or do not report TB symptoms. The relationship between human immunodeficiency virus (HIV) and subclinical TB is not yet well understood. We estimated the prevalence of subclinical pulmonary TB in household contacts of index TB patients in two South African provinces, and how this differed by HIV status.

**Methods:**

This was a cross-sectional, secondary analysis of baseline data from the intervention arm of a household cluster randomised trial. Prevalence of subclinical TB was measured as the number of household contacts aged ≥ 5 years who had positive sputum TB microscopy, culture or nucleic acid amplification test (Xpert MTB/Rif or Xpert Ultra) results on a single sputum specimen and who did not report current cough, fever, weight loss or night sweats on direct questioning. Regression analysis was used to calculate odds ratios (OR) and 95% confidence intervals (CI) for the association between HIV status and subclinical TB; adjusting for province, sex and age in household contacts; and HIV status in index patients.

**Results:**

Amongst household contacts, microbiologically confirmed prevalent subclinical TB was over twice as common as symptomatic TB disease (48/2077, 2.3%, 95% CI 1.7–3.1% compared to 20/2077, 1.0%, 95% CI 0.6–1.5%). Subclinical TB prevalence was higher in people living with HIV (15/377, 4.0%, 95% CI 2.2–6.5%) compared to those who were HIV-negative (33/1696, 1.9%, 95% CI 1.3–2.7%; *p* = 0.018). In regression analysis, living with HIV (377/2077, 18.2%) was associated with a two-fold increase in prevalent subclinical TB with 95% confidence intervals consistent with no association through to a four-fold increase (adjusted OR 2.00, 95% CI 0.99–4.01, *p* = 0.052). Living with HIV was associated with a five-fold increase in prevalent symptomatic TB (adjusted OR 5.05, 95% CI 2.22–11.59, *p* < 0.001).

**Conclusions:**

Most (70.6%) pulmonary TB diagnosed in household contacts in this setting was subclinical. Living with HIV was likely associated with prevalent subclinical TB and was associated with prevalent symptomatic TB. Universal sputum testing with sensitive assays improves early TB diagnosis in subclinical household contacts.

**Supplementary Information:**

The online version contains supplementary material available at 10.1186/s44263-023-00022-5.

## Background

Approximately 10.6 million people developed tuberculosis (TB) disease in 2021, resulting in 1.6 million deaths and making it the second leading cause of death from a single infectious disease worldwide after COVID-19 [1]. Whilst TB was historically divided into binary states (‘latent TB’ and ‘active TB disease’), in recent years, there has been increased recognition that this dichotomisation ignores a wide spectrum, during which symptomatology, pathology and microbiological findings may vary considerably and may progress or regress [2]. Nomenclature and definitions vary; however, subclinical TB is generally accepted to be defined by microbiologically or radiographically confirmed TB in the absence of TB symptoms and with the potential to cause transmission [2–6]. People with subclinical TB are thus of high clinical and public health importance, as early detection and treatment may improve health outcomes and prevent transmission.

A systematic review of national prevalence surveys in Africa and Asia suggests that 50.4% (interquartile range [IQR] 39.8–62.3%) of TB is subclinical [7]. National prevalence surveys often use TB symptom screens to identify individuals who may have TB. However, these screens typically define symptomatic TB as having a cough for 2 weeks or longer, which may exclude people with less noticeable or shorter duration symptoms. Additionally, most prevalence surveys have an initial symptom and/or chest X-ray screening step; thus, people with radiologically negative subclinical TB would not be investigated by sputum microbiological tests. This includes South Africa’s first national TB prevalence survey which found a high proportion of prevalent TB to be subclinical (57.7%), despite using a more sensitive symptom screen incorporating cough (persistent of any duration), drenching night sweats, unexplained weight loss and unexplained fever for at least 2 weeks [8, 9]. Additional studies in South Africa have reported high estimates of subclinical TB as a proportion of microbiologically confirmed TB, including 44.7% in a secondary analysis of trial data in a high HIV/TB prevalence setting, 77.6% in a rural community setting in KwaZulu-Natal province and 54.6% in targeted universal TB testing in high-risk groups attending primary care facilities [10–12].

There is little data describing the prevalence of subclinical TB in household contacts of people with TB, despite the known high prevalence of TB in this group [13]. Estimates of the proportion of asymptomatic TB amongst all household contacts with TB vary from 50 to 96% across different settings [14–16]. Over time, changing awareness of TB symptoms and improved case detection for symptomatic patients may alter proportions of symptomatic and subclinical TB disease, necessitating an update of this estimate in the South African setting.

HIV is an important risk factor for developing symptomatic TB disease, [1, 17] but the relationship between subclinical TB and HIV has not been fully explored. Typical TB symptoms are less common in people coinfected with HIV and TB [18–20]. A better understanding of the relationship between subclinical TB and HIV is therefore important to inform screening strategies for people with and without HIV.

In this study, we determined the prevalence of subclinical pulmonary TB in household TB contacts in two South African provinces, and the association of subclinical pulmonary TB with HIV status.

## Methods

### Data source

This was a cross-sectional, secondary analysis of baseline data from a previously completed household cluster-randomised controlled trial (ISRCTN16006202), for which the protocol and results are published in full elsewhere [21, 22]. The dataset and the code used for this study have been deposited in the LSHTM Data Compass repository (https://doi.org/10.17037/DATA.00003373). The trial compared two strategies for the management of household contacts of people with microbiologically confirmed TB: intensified home-based TB and HIV screening, and a referral letter strategy. The present study used the baseline data from the intervention arm of the trial only, where household contact participants were offered investigation for HIV and TB regardless of symptoms. The Strengthening the Reporting of Observational Studies in Epidemiology (STROBE) reporting standards were followed (Additional file 1).

### Participants and setting

Data collection for the original trial took place between December 2016 and March 2019 in two South African provinces: Mangaung Municipality in Free State Province and Capricorn District in Limpopo Province. HIV prevalence was 17.0% and 10.9% in Free State and Limpopo provinces, respectively, in 2017 [23]. TB incidence rates were 616 per 100,000 and 328 per 100,000 in Mangaung and Capricorn respectively in 2015 [24, 25].

Index patients with TB were identified from government medical facilities, with those aged 7 years or above required to have microbiologically confirmed pulmonary TB for inclusion, while those under seven could have TB of any form (microbiologically confirmed or clinically diagnosed) if diagnosed by a physician. Index patients could be living, or recently deceased, and were required to have been diagnosed with TB in the 6 weeks prior to recruitment. We excluded index patients who were incarcerated or in long-term inpatient care. Household contacts of index patients were defined as people living together within a set of rooms under a contiguous roof, linked by doorways and windows, through which air moved, and where household members had shared airspace by either sleeping overnight at least once, or had shared at least two meals in the same household as the index patient in the 14 days prior to the index patient’s diagnosis of TB [21, 22]. Those who did not meet this definition or who did not consent to participate were excluded. Household contacts under five were excluded from the present analysis as they were not required to produce sputum. There were no other exclusion criteria for household contacts or index cases, as described in the protocol for the original trial [22].

### Procedures and case definitions

Within 14 days of recruitment of index patients randomly allocated to the intensive screening arm, a home visit was undertaken where household contacts received: a questionnaire covering socio-demographic characteristics, risk factors for TB, presence of symptoms, self-reported diabetes and history of previous TB and HIV treatment; point of care HIV testing, followed by measurement of CD4 count if positive; and those older than five were requested to produce a single sputum specimen regardless of their report of TB symptoms. This specimen was subjected to microbiological TB testing, including nucleic acid amplification test (NAAT with Xpert MTB/RIF or Xpert Ultra, Cepheid, Sunnyvale, USA), smear microscopy and mycobacterial growth indicator tube (MGIT) culture. All were asked to rinse their mouths with water prior to sputum collection. If the household contact was unable to produce a mucoid sputum specimen, we requested them to cough repeatedly and then spit whatever was in their mouth into the container and repeat this until ≥ 3 ml was collected. The study laboratories were the public sector National Health Laboratory Service (NHLS) which has its own internal quality assurance programmes. At the time of the trial, South Africa was changing over Xpert MTB/RIF to Xpert MTB/RIF Ultra cartridges and the changeover differed at each site’s NHLS laboratory.

Microbiologically confirmed pulmonary TB was defined as a household contact with a positive sputum auramine stained microscopy (scanty or any + to +  +  + positive), or positive NAAT, or culture result speciated as *M tuberculosis* complex detected. TB symptoms were defined as the presence of any duration of cough, weight loss, night sweats or fever [26]. ‘Symptomatic pulmonary TB’ was defined by microbiologically confirmed TB in a participant who reported at least one of these four TB symptoms. ‘Subclinical pulmonary TB’ was defined by microbiologically confirmed pulmonary TB, in the absence of all four TB symptoms.

### Statistical analysis

Data was analysed using R (version 4.1.2, R Core Development Team). We used descriptive statistics to summarise characteristics of household contacts, stratified by TB status. To assess for non-response bias, characteristics of household contacts included in the analysis were compared with those identified by index patients in the intervention arm at baseline, but who did not participate, and between contacts with and without microbiological TB results.

Prevalence of subclinical and symptomatic pulmonary TB was calculated as the number of cases in household contacts divided by the total number of household contacts for whom a microbiological TB result was available, with Clopper Pearson 95% confidence intervals (CIs) [27, 28]. We also stratified prevalence estimates by province, HIV status and sex and compared TB status using a two-proportion *Z* test [29].

**Fig. 1:**
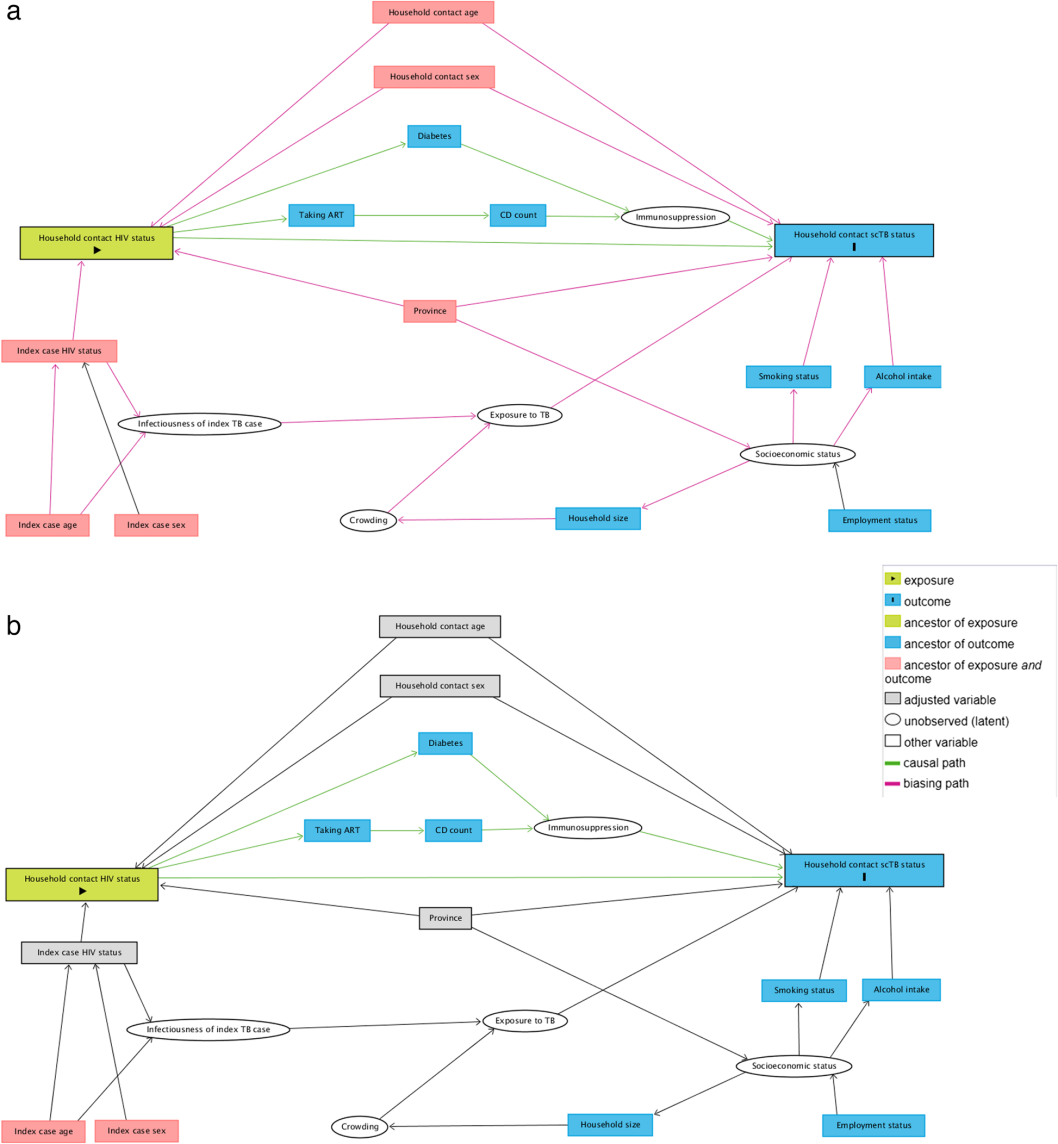
Directed acyclic graph demonstrating a putative causal relationship between HIV status and subclinical TB status (unadjusted and adjusted). Arrows represent directional relationships between variables. Created using DAGitty version 3.0. ART antiretroviral therapy, CD4 cluster differentiation four T-lymphocyte, HIV human immunodeficiency virus, scTB subclinical tuberculosis

For regression analysis, missing data was excluded listwise where missingness was less than 5%, with the assumption that data was missing at random [30]. We planned that multiple imputation would be considered where an independent variable for regression analysis had a missingness of 5% or more, though this was not required. To investigate the association between HIV status and TB disease state, univariable and multivariable binomial logistic regression models were constructed to estimate unadjusted and adjusted ORs and 95% CIs for two outcome comparisons: (1) subclinical TB versus no TB and (2) symptomatic TB versus no TB. To allow for clustering, the province of residence was included as a fixed effect, and robust standard errors were used to account for household clustering [31, 32]. For multivariable models, a minimally sufficient adjustment set of covariates consisting of household contact age (continuous), household contact sex, province of residence and index case HIV status were identified through a directed acyclic graph (DAG) demonstrating putative pathways of association between the exposure of HIV status and the outcome of subclinical TB (Fig. 1) [24, 33, 34].

A sensitivity analysis was conducted by excluding TB patients that were positive on smear only with no confirmation from other microbiological methods such as NAAT or culture.

## Results

### Participant characteristics

**Fig. 2:**
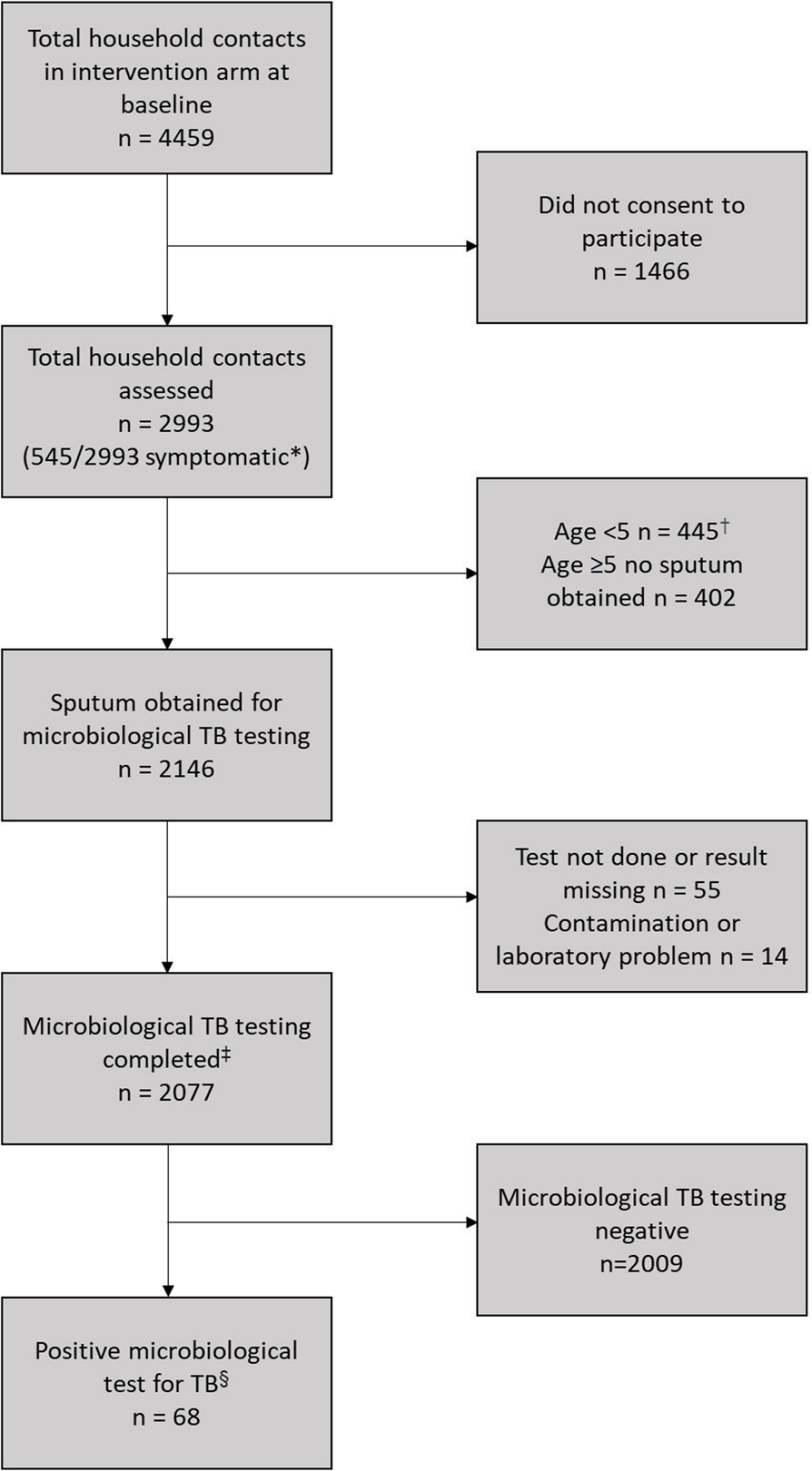
Microbiological TB testing participant flow diagram. TB tuberculosis, n number. *Symptomatic = current cough, fever, weight loss or night sweats. ^†^Under 5s were excluded as they were not required to produce a sputum sample. ^‡^Result available for nucleic acid amplification test (NAAT) and/or sputum smear and/or sputum culture. ^§^For at least one of nucleic acid amplification test (NAAT) and/or sputum smear and/or sputum culture

**Fig. 3:**
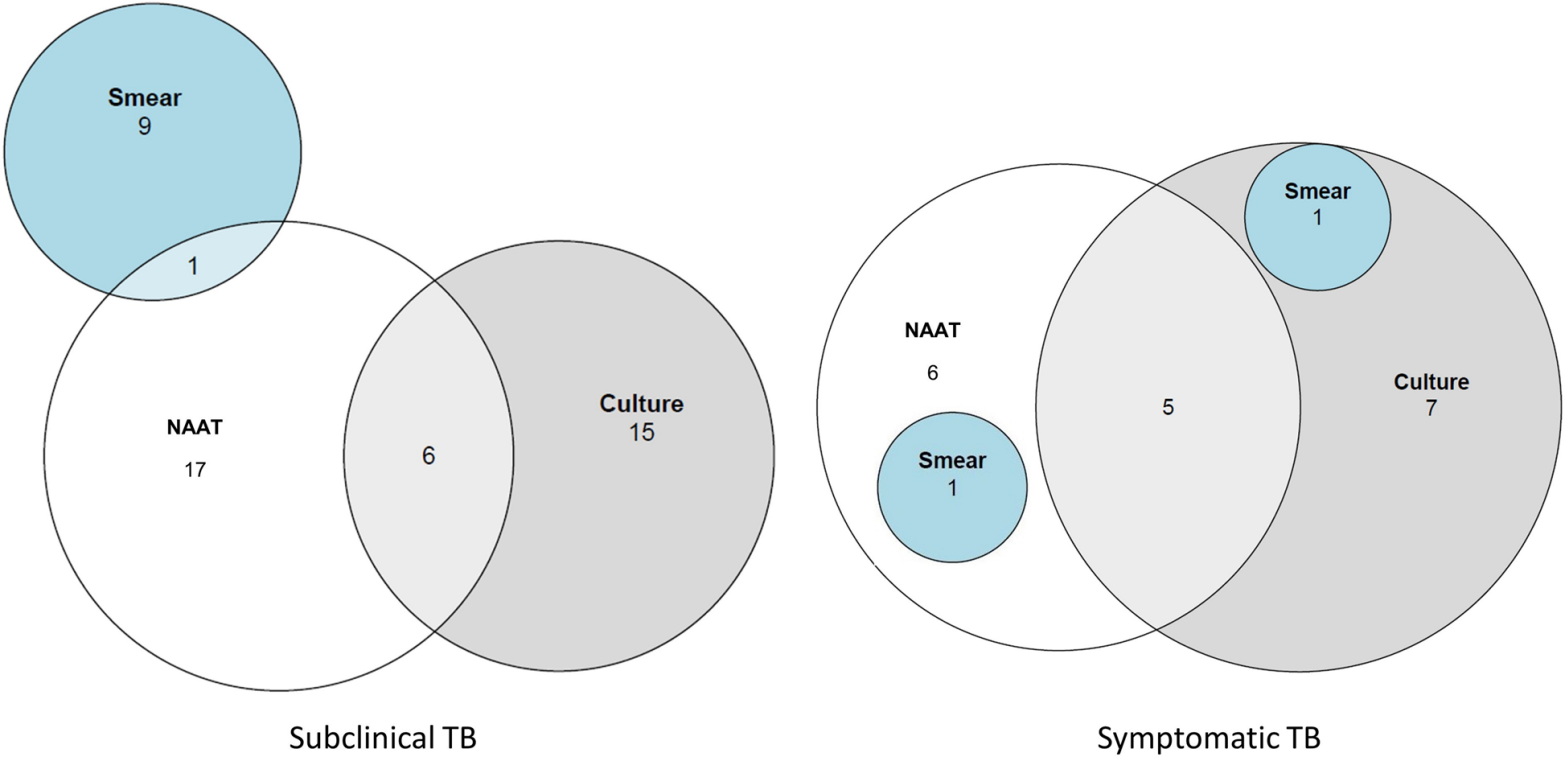
Microbiological methods of TB diagnosis. NAAT nucleic acid amplification test, TB tuberculosis. Includes all participants diagnosed on smear and/or NAAT and/or culture

A total of 4459 household contacts of 1032 index TB patients randomised to the intervention were identified, of whom 2993 (67.1%) household contacts from 923 households consented to participate. 445 household contacts under 5 years of age were excluded from these analyses as those under 5 were not required to produce sputum. Sputum was obtained for microbiological TB testing in 2146/2548 (84.2%) of the household contact participants aged ≥ 5 from which a result was available for at least one microbiological test (NAAT, smear or culture) for 2077 (81.5%) household contacts from 853 households (Fig. 2). For participants with a microbiological TB result, 95.3% (1979/2077) had a NAAT result, 73.4% (1525/2077) had a smear result, and 76.4% (1586/2077) had a culture result. For participants classified as having subclinical and symptomatic TB, 85.4% (41/48) and 65.0% (13/20), respectively, were diagnosed on a single microbiological test (Fig. 3).

Table 1 provides characteristics of the 2077 household contact participants with a microbiological TB result, of whom 1190 (57.3%) were residents in Mangaung and 887 (42.7%) in Capricorn. The median age of household contacts with a sputum result was 24 years (IQR 13–46), and 64.9% were female. 18.2% were living with HIV, of whom 66.0% were taking antiretroviral therapy (ART). Missing values were common for the variables of CD4 count (80.4%) and ART status (23.9%) in household contacts living with HIV. There were low levels (< 5%) of missing data for the remainder of the variables (Table 1). Characteristics of the 853 index patients are available in Table S1 (Additional file 2).

**Table 1:** Demographic, socioeconomic and clinical characteristics of household contacts, stratified by TB status

	**Total** *N* = 2077	**No microbiological TB** *N* = 2009	**Subclinical TB** *N* = 48	**Symptomatic TB** *N* = 20
Overall sample (%)	100.0	96.7	2.3	1.0
Province (*N*, %)				
Mangaung	1190 (57.3)	1149 (57.2)	28 (58.3)	13 (65.0)
Capricorn	887 (42.7)	860 (42.8)	20 (41.6)	7 (35.0)
Sex (*N*, %)				
Male	729 (35.1)	710 (35.3)	12 (25.0)	7 (35.0)
Female	1348 (64.9)	1299 (64.7)	36 (75.0)	13 (65.0)
Age (years) [median, IQR]	24 (13–46)	24 (13–46)	27 (13–45)	44 (23–58)
Age group (*N*, %)				
5–9 years	254 (12.2)	247 (12.3)	6 (12.5)	1 (5.0)
10–15 years	420 (20.2)	410 (20.4)	8 (16.7)	2 (10.0)
16–24 years	379 (18.2)	368 (18.3)	8 (16.7)	3 (15.0)
25–34 years	286 (13.8)	274 (13.6)	10 (20.8)	2 (10.0)
35–44 years	187 (9.0)	182 (9.1)	3 (6.3)	2 (10.0)
45–54 years	180 (8.7)	171 (8.5)	6 (12.5)	3 (15.0)
55–64 years	183 (8.8)	175 (8.7)	3 (6.3)	5 (25.0)
65 + years	188 (9.1)	182 (9.1)	4 (8.3)	2 (10.0)
Employment status				
Currently employed	169 (8.1)	164 (8.2)	3 (6.3)	2 (10.0)
Not employed	818 (39.4)	785 (39.1)	21 (43.8)	12 (60.0)
Student	824 (39.7)	806 (40.1)	14 (29.2)	4 (20.0)
Other	263 (12.7)	251 (12.5)	10 (20.8)	2 (10.0)
Missing	3 (0.1)	3 (0.1)	0	0
Average household size (mean, ± SD)	4.5 (2.6)	4.5 (2.6)	5.0 (3.2)	1.8 (1.0)
HIV status (*N*, %)				
Positive	377 (18.2)	352 (17.5)	15 (31.3)	10 (50.0)
Negative	1696 (81.7)	1653 (82.3)	33 (68.8)	10 (50.0)
Missing	4 (0.2)	4 (0.2)	0	0
Currently taking ART if HIV-positive (*N*, %)				
Yes	249 (66.0)	230 (65.3)	12 (80.0)	7 (70.0)
No	38 (10.1)	36 (10.2)	1 (6.7)	1 (10.0)
Missing	90 (23.9)	86 (24.4)	2 (13.3)	2 (20.0)
CD4 count if HIV-positive (*N*, %) [cells/mm^3^]				
< 200	11 (2.9)	11 (3.1)	0	0
200–499	30 (8.0)	29 (8.2)	0	1 (10.0)
500 +	33 (8.8)	32 (9.1)	0	1 (10.0)
Missing	303 (80.4)	280 (79.5)	15 (100.0)	8 (80.0)
Diabetes (*N*, %)				
Yes	39 (1.9)	36 (1.8)	2 (4.2)	1 (5.0)
No	2038 (98.1)	1973 (98.2)	46 (95.8)	19 (95.0)
Smoking status (*N*, %)				
Current smoker	228 (11.0)	217 (10.8)	6 (12.5)	5 (25.0)
Previously smoked	39 (1.9)	38 (1.9)	0	1 (5.0)
Never smoked	1808 (87.0)	1752 (87.2)	42 (87.5)	14 (70.0)
Missing	2 (0.1)	2 (0.1)	0	0
Currently drinks alcohol (*N*, %)				
Yes	326 (15.7)	308 (15.3)	10 (20.8)	8 (40.0)
No	1749 (84.2)	1699 (84.6)	38 (79.2)	12 (60.0)
Missing	2 (0.1)	2 (0.1)	0	0

Percentages may not total 100 due to rounding (one decimal place). Age rounded to whole year. *ART* antiretroviral therapy, *CD4* cluster differentiation four T-lymphocytes measured as cells per cubic millimetre of blood, *HIV* human immunodeficiency virus, *IQR* interquartile range, *N* number, *SD* standard deviation, *Missing data* 0 for each variable unless otherwise specified

From household census information available for the 4459 household contacts identified in the intervention arm of the trial at baseline, the median age was similar compared to the 2993 household contacts that participated in the study (19 years, IQR 3–37 and 17 years, IQR 8–37, respectively). 58.0% of the household contacts were female compared with 61.8% of those who opted to participate. Table S2 (Additional file 2) shows characteristics of participants who did and did not have sputum results, restricted to age ≥ 5. The proportion of participants with a sputum result available did not appear to vary notably between study sites (Capricorn: 887/1106, 80.2%; Mangaung: 1190/1442, 82.5%). Household contact participants without a sputum result were younger than those with one (median age 10 years, IQR 6–24). In keeping with the younger age range, the proportion of participants living with HIV, currently smoking and drinking alcohol were also lower amongst those without a sputum result (Additional file 2: Table S2).

When stratified by an individual interviewer, the proportion of TB patients that were classified as subclinical based on the absence of cough, weight loss, night sweats and fever ranged from 50 to 100%, although numbers were small (range: 1–14 subclinical patients identified from 11 interviewers, with a further 16 interviewers identifying no subclinical patients; Additional file 2: Table S3). There was no evidence to support a difference in subclinical TB classification between interviewers (Fisher’s exact, *p* = 0.98).

### Prevalence of subclinical and symptomatic TB

**Fig. 4:**
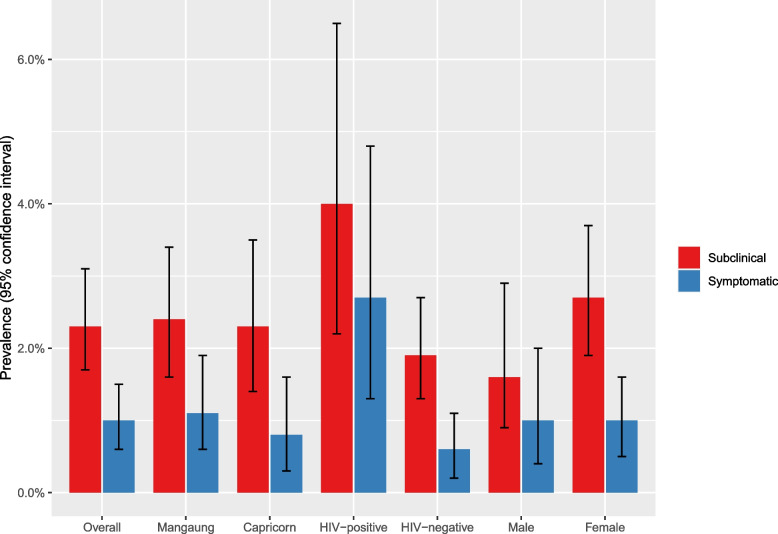
Prevalence of subclinical and symptomatic TB amongst household contacts with a TB sputum result. Confidence intervals were calculated using the Clopper-Pearson method. Data on HIV status missing for 4/2097 participants

The overall prevalence of pulmonary TB in household contacts was 68/2077 (3.3%, 95% CI 2.6–4.1%). Prevalence of subclinical pulmonary TB in household contacts was 48/2077 (2.3%, 95% CI 1.7–3.1%) and prevalence of symptomatic pulmonary TB was 20/2077 (1.0%, 95% CI 0.6–1.5%) (Fig. 4). Subclinical pulmonary TB was 2.4 times as common as symptomatic pulmonary TB, with 70.6% (48/68) having subclinical TB (95% CI 58.3–81.0%).

The prevalence of subclinical TB (Mangaung: 28/1190, 2.4%, 95% CI 1.6–3.4%; Capricorn: 20/887, 2.3%, 95% CI 1.4–3.5%, *p* = 0.883) and symptomatic TB (Mangaung: 13/1190, 1.1%, 95% CI 0.6–1.9%; Capricorn: 7/887, 0.8%, 95% CI 0.3–1.6%, *p* = 0.484) were similar across study sites. Subclinical and symptomatic TB prevalence was higher in contacts living with HIV (15/377, 4.0%, 95% CI 2.2–6.5% and 10/377, 2.7%, 95% CI 1.3–4.8%, respectively) compared to HIV-negative (33/1696, 1.9%, 95% CI 1.3–2.7%, *p* = 0.018 and 10/1696, 0.6%, 95% CI 0.2–1.1%, *p* < 0.001, respectively). Subclinical TB prevalence was higher amongst women (36/1348, 2.7%, 95% CI 1.9–3.7%) than men (12/729, 1.6%, 95% CI 0.9–2.9%, *p* = 0.014). There was a similar prevalence of symptomatic TB amongst women (13/1348, 1.0%, 95% CI 0.5–1.6%) and men (7/729, 1.0%, 95% CI 0.4–2.0%, *p* = 0.987).

### Association between HIV status and TB state

In adjusted analysis, living with HIV was likely associated with a two-fold increase in prevalent subclinical TB (adjusted OR 2.00, 95% CI 0.99–4.01, *p* = 0.052). 95% confidence intervals for this effect estimate were consistent with no association through to a four-fold increase in prevalent subclinical TB. Living with HIV was associated with a five-fold increase in prevalent symptomatic TB, with 95% confidence intervals consistent with between a 2- and 12-fold increase (adjusted OR 5.05, 95% CI 2.22–11.59, *p* < 0.001) (Table 2). Detailed model results with additional covariates are available in Tables S4 and S5 (Additional file 2).

**Table 2:** Association between HIV status and TB state assessed by univariable and multivariable logistic regression

Analysis	HIV status	Number/sample	Unadjusted OR (95% CI) [*p* value]	Adjusted^*^ OR (95% CI) [*p* value]
Subclinical TB versus no TB^a^	Negative	33/1686	Ref	Ref
	Positive	15/367	2.15 (1.15–4.01) [0.016]	2.00 (0.99–4.01) [0.052]
Symptomatic TB versus no TB^b^	Negative	10/1663	Ref	Ref
	Positive	10/362	4.61 (1.94–11.13) [< 0.001]	5.05 (2.22–11.59) [< 0.001]

*CI* confidence interval, *OR* odds ratio, *Ref* reference category. Unadjusted ORs calculated using simple logistic regression via the glm() function in R, with province included as fixed term effect to adjust for clustering. Adjusted ORs were calculated using multiple logistic regression via the glm() function in R. Robust-clustered CIs were calculated based on clustering at a household level, using the coeftest() function in R. ^*^Adjusted for the province, household contact sex, household contact age and index case HIV status. Missing data dealt with listwise: ^a^1955/2057 included in the adjusted analysis, ^b^1929/2029 included in the adjusted analysis

### Sensitivity analysis

A sensitivity analysis was conducted by excluding TB patients who were positive on sputum smear only, with no confirmation from NAAT or culture. Nine TB patients were excluded on this basis, of which 2/9 were people living with HIV. This resulted in an overall prevalence of pulmonary TB in household contacts of 59/2077 (2.8%, 95% CI 2.2–3.6%), a subclinical pulmonary TB prevalence of 39/2077 (1.9%, 95% CI 1.3–2.6%) and a symptomatic pulmonary TB prevalence remaining at 20/2077 (1.0%, 95% CI 0.6–1.5%). In sensitivity analysis, subclinical TB accounted for 66.1% (39/59) of TB patients amongst household contacts. Living with HIV was associated with subclinical TB in adjusted analysis with an effect estimate of similar magnitude to that presented in the main results (adjusted OR 2.30, 95% CI 1.05–5.00, *p* = 0.037) (Additional file 2: Table S6).

## Discussion

In this secondary analysis of baseline data from a household-randomised controlled trial in two provinces of South Africa, we found that the prevalence of subclinical TB in household contacts of index TB patients substantially exceeded the prevalence of symptomatic TB. The yield of TB diagnosis in household contacts (3.3%) was comparable with systematic review estimates of microbiologically confirmed TB prevalence amongst household contacts (3.2%, 95% CI 2.6–3.7%), and a smaller-scale study carried out in an alternative geographic location in Limpopo (3.9%, 95% CI 2.0–6.9%) [35–37]. The high percentage of TB patients in household contacts that were subclinical (70.6%, 95% CI 58.3–81.0%) exceeds that estimated through a systematic review of country prevalence surveys (50.4%), South Africa’s recent prevalence survey (57.7%), a secondary analysis of trial data (44.7%) and adult caregiver contacts of children with TB (43.5%) [7, 8, 10, 38]. However, it is in keeping with estimates from a smaller-scale study in a rural South African clinic (70.0%) and community (77.6%) setting [11]. Cross-sectional data from India and China has found the proportion of asymptomatic TB in household contacts of index TB patients to be 96.6% and 50.0%, respectively [14, 15]. However, for the former, a definition of asymptomatic was not provided and for the latter absence of symptoms was not required for subclinical TB diagnosis. HIV prevalence is also likely to have differed significantly from the South African context. An evaluation of active case finding for TB and HIV in household contacts of index TB patients in South Africa found 151/169 (89.3%) of TB patients in household contacts were asymptomatic for cough, fever, weight loss and night sweats in 2009 [16]. Our estimate suggests the subclinical proportion has reduced since that time, but still accounts for the majority of TB disease burden in household contacts in this setting. Differences in microbiological endpoints for TB diagnosis may also account for differences in estimates of subclinical TB prevalence, for example through increased reliance on smear positivity in earlier studies which is less sensitive and specific for TB diagnosis [16]. In sensitivity analysis where TB patients diagnosed by smear alone were excluded in case of false positive results, the majority of pulmonary TB amongst household contacts was still subclinical (39/59, 66.1%).

The higher proportion of subclinical TB in the present study compared to national prevalence surveys can be partially explained by the detection of people with asymptomatic, radiography-negative, microbiologically positive TB that are missed in surveys that rely on an initial symptom and chest X-ray screen to determine eligibility for sputum testing [7, 8]. However, the use of a four-symptom screen for TB in the present study should reduce the proportion of people categorised as having subclinical TB compared to many national prevalence surveys that use the less sensitive cough ≥ 2 weeks screen [7, 36]. Unlike surveillance studies, in our study, household contacts had recently been exposed to an infectious person with TB and therefore may be earlier in their disease trajectory predisposing to the subclinical phase. The robustness of symptom screening by interviewers is another factor which can influence the proportion of people with TB categorised as subclinical. In our study, there was no evidence to suggest that detection of symptoms in contacts varied by interviewer. In addition, the success of programmes to detect and treat symptomatic pulmonary TB will increase the remaining proportion of TB which is subclinical through the reduction of symptomatic patients in estimates. Nevertheless, our findings are broadly consistent with the literature in estimating that a high proportion of people with TB in the community are subclinical. This has important implications for household screening interventions, which if reliant on symptom-based screening will miss a significant proportion of prevalent infectious TB. Household contacts should undergo more intensive investigation for TB, irrespective of symptoms. This will achieve earlier diagnosis, though evidence for reduced mortality with TB screening versus passive case-finding in the general population and at-risk groups has not been conclusively established via the latest systematic review [39]. However, in many of the included studies, a positive symptom screen was required for further TB testing, ruling out the detection of people with subclinical TB. More intensive TB screening is consistent with World Health Organization (WHO) guidance on household contact screening, though can be difficult to implement in high TB burden settings due to resource constraints [40]. Recent cluster-randomised trial evidence found a referral letter strategy for screening of household contacts was equivalent to intensive HIV and TB screening, regarding outcomes of incident TB or death [21]. This may present a pragmatic solution.

Our results suggest a two-fold increase in the risk of prevalent subclinical TB in those who are living with HIV compared to those who are not, though the confidence intervals are compatible with no increased risk through to a four-fold increased risk. We also found that living with HIV was associated with a five-fold increase in the risk of prevalent symptomatic TB, with confidence intervals compatible with two-fold to 12-fold increased risk. The magnitude of the latter association appeared greater. However, the overall higher burden of subclinical TB in our sample when compared with symptomatic TB meant that the majority of TB patients amongst those with HIV were subclinical. These findings could potentially be explained by the influence of immunosuppression. There is evidence that people living with HIV who have symptomatic TB have lower CD4 counts than those with subclinical TB [41, 42]. Those with HIV-related immunosuppression may move more rapidly through the subclinical phase towards symptomatic TB [43, 44]. An alternative explanation for the findings is that those with HIV may have a greater awareness of TB symptoms due to frequently being screened for them at HIV care appointments and therefore may be more likely to say yes when asked about them resulting in a symptomatic TB classification. This seems less likely given it does not account for the differences in CD4 count between subclinical and symptomatic TB groups presented in other works [41, 42].

In a previous study, symptomatic TB disease was more prevalent than subclinical TB disease amongst a cohort of 654 ART-naïve participants with HIV, using an equivalent symptom screen to the present study [41]. We found subclinical TB disease was more prevalent than symptomatic TB disease in those living with HIV, though the proportion of symptomatic TB was greater than for the HIV-negative group. This may be explained by ART usage. In our study, 66.0% of participants living with HIV were currently taking ART. If HIV-related immunosuppression accelerates progression to symptomatic TB, taking ART should to an extent mitigate that process through suppression of viral load and reconstitution of CD4 counts [43]. This would account for a greater proportion of subclinical TB in participants living with HIV in our study compared to those who are ART naïve [41]. Reliance on symptom screening in people living with HIV will miss opportunities for earlier TB case detection. Immunosuppression from HIV may encourage faster progression from subclinical to symptomatic TB [41, 42]. If so, widespread ART usage and consequent viral suppression may further increase the proportion of subclinical TB disease, reducing the effectiveness of TB symptom screens in this group over time.

Our study found that subclinical TB in household contacts was more prevalent amongst women than men, whilst symptomatic TB prevalence was similar amongst women and men. It is well established that overall TB prevalence is higher amongst men; [45] however, evidence for an association between subclinical TB and sex is limited and mixed [46–48]. This was not the primary focus of our study; however, further work is needed to explore potential sex and gender-related differences in TB presentation, including cultural norms around symptom reporting.

Strengths of the study include the completeness of data collection for household contact HIV status (the exposure of interest), as well as the availability of data for key confounders that were mapped using a DAG. The creation of the DAG inevitably involves some subjectivity but makes explicit the assumptions made when selecting potential confounders. The study also had several limitations. A high level of missingness for data on CD4 count and ART status in household contacts precluded further analysis of the relationship between immunosuppression and subclinical TB, which is an important area for future work, alongside exploration of additional potential risk factors for subclinical TB such as smoking status. Sputum screening was also incomplete. It is generally challenging to collect sputum samples from people in the community as some will struggle to produce a sample, especially children [49]. The sputum collection rate achieved in our study was comparable to other studies in community and household settings [16, 50]. The younger median age of those with no sputum result could bias results in the direction of an underestimate of subclinical TB prevalence, given previous evidence of an association between younger age and subclinical TB [46, 47]. The changeover from Xpert MTB/RIF to Xpert MTB/RIF Ultra cartridges in South Africa part way through this study prevented further analysis of the relationship between the lowest ‘trace’ semiquantitative detection of the Ultra system and subclinical TB; however, this should be explored in future work.

The *p* value and 95% CI for the adjusted estimate of the association between living with HIV and subclinical TB marginally crossed the threshold typically assigned as statistically non-significant. We have avoided arbitrary dichotomisation of the findings using these thresholds, instead discussing the potential implications of the odds ratios and corresponding 95% CIs [51]. Although the 95% CI included no effect, the likelihood of a true association is further supported by results from a sensitivity analysis where TB patients who were diagnosed on smear alone were excluded in case of false positives (Additional file 2: Table S6). An effect estimate of similar magnitude was obtained for the association between living with HIV and subclinical TB, with upper and lower 95% confidence interval limits consistent with an association of 1- to fivefold magnitude (adjusted OR 2.30, 95% CI 1.05–5.00, *p* = 0.037).

The generalisability of our findings outside of South Africa may be limited given the unique epidemiological context with a significant dual burden of HIV and TB disease [9]. In addition, the present study only looked at point-prevalent TB. There is potential for subclinical TB to be ‘unmasked’ as symptomatic with symptom development over time, especially if the duration of time spent in the subclinical phase of TB disease is short. Further, our ability to detect paucibacillary TB patients was limited by a lack of induced sputum or repeated sputum sampling. Nevertheless, to our knowledge, the current study presents the most up-to-date estimate of the prevalence of subclinical TB in household contacts of index TB patients in a South African setting. A priority for future research should be establishing the effectiveness, safety and cost-effectiveness of direct microbiological screening for TB in at-risk groups, compared with existing strategies of symptom screens with or without chest X-rays. Groups of particular interest are household TB contacts and people living with HIV.

## Conclusions

We found that the prevalence of subclinical pulmonary TB in household contacts of index TB patients in two South African provinces substantially exceeded the prevalence of symptomatic pulmonary TB. Living with HIV was likely associated with a two-fold increase in prevalent subclinical TB and was associated with a five-fold increase in prevalent symptomatic TB. To improve early TB diagnosis in household contacts, more sensitive screening approaches may be needed such as universal sputum examination. Identifying and treating people living with subclinical TB should be a focus of future TB control efforts. This would ensure initiation of TB treatment earlier in the course of the disease and is likely to reduce morbidity and mortality due to TB and importantly prevent TB transmission.

### Supplementary Information

**Additional file 1.** STROBE Checklist. Contains a completed Strengthening the Reporting of Observational studies in Epidemiology (STROBE) checklist.

**Additional file 2.** Contains supplementary data and analyses in tables S1-6 as follows: **Table S1.** Demographic and clinical characteristics of index cases. **Table S2.** Comparison of household contact characteristics between those with and without a microbiological TB result. **Table S3.** Proportion of TB patients classified as subclinical based on individual interviewer symptom screens. **Table S4.** Association of HIV status with subclinical TB versus no TB assessed by multiple logistic regression (full model). **Table S5.** Association of HIV status with symptomatic TB versus no TB assessed by multiple logistic regression (full model). **Table S6.** Sensitivity analysis.

## Data Availability

The dataset and the code used for this study have been deposited in the LSHTM Data Compass repository (available at: https://doi.org/10.17037/DATA.00003373). The code is freely available to download. The dataset can be requested from Emily Webb and Neil Martinson via the ‘request access button’ in the provided link; however, the release of the data is subject to approval from the Institutional Review Board of the original trial and completion of a Data Transfer Agreement is required.

## Abbreviations

ART: Antiretroviral therapy
CD4: Cluster differentiation four T-lymphocyte
CI: Confidence interval
DAG: Directed acyclic graph
IQR: Interquartile range
HIV: Human immunodeficiency virus
MGIT: Mycobacterial growth indicator tube
MTB: Mycobacterium tuberculosis
NAAT: Nucleic acid amplification testing
OR: Odds ratio
SD: Standard deviation
TB: Tuberculosis
WHO: World Health Organization
