# Supplementary material for: Prevalence of subclinical pulmonary tuberculosis and its association with HIV in household contacts of index tuberculosis patients in two South African provinces: a secondary, cross-sectional analysis of a cluster-randomised trial
Source: BMC Glob Public Health. 2023 Nov 1;1:21. doi: 10.1186/s44263-023-00022-5 (PMC11116238; doi:10.1186/s44263-023-00022-5)
Supplement: Supplementary file 1 — Additional file 1. STROBE Checklist. Contains a completed Strengthening the Reporting of Observational studies in Epidemiology (STROBE) checklist. [file 44263_2023_22_MOESM1_ESM.docx]

STROBE Statement—checklist of items that should be included in reports of observational studies

|  | Item No. | Recommendation | Page  No. | Relevant text from manuscript |
| --- | --- | --- | --- | --- |
| **Title and abstract** | 1 | (*a*) Indicate the study’s design with a commonly used term in the title or the abstract | 1 | Prevalence of subclinical pulmonary tuberculosis and its association with HIV in household contacts of index tuberculosis patients in two South African provinces: a secondary, cross-sectional analysis of a cluster-randomised trial |
|  |  | (*b*) Provide in the abstract an informative and balanced summary of what was done and what was found | 2 | Abstract  Background: People with subclinical tuberculosis (TB) have microbiological evidence of Mycobacterium tuberculosis disease caused by Mycobacterium tuberculosis, but either do not have, or do not report TB symptoms. The relationship between human immunodeficiency virus (HIV) and subclinical TB is not yet well understood. We set out to estimated the prevalence of subclinical pulmonary TB in household contacts of index TB patients in two South African provinces, and how this differed by HIV status.  Methods: This was a cross-sectional analysis of baseline data from the intervention arm of a household cluster randomised trial. Prevalence of subclinical TB was measured as the number of household contacts aged ≥5 years who had positive sputum TB microscopy, culture, or nucleic acid amplification test (Xpert MTB/Rif or Xpert Ultra) results on a single sputum specimen and who did not report current cough, fever, weight loss or night sweats on direct questioning. Regression analysis was used to calculate odds ratios (OR) and 95% confidence intervals (CI) for the association between HIV status and subclinical TB, adjusting for province, sex, and age and HIV status in household contacts, and HIV status in index patients.  Results: Among household contacts, microbiologically-confirmed prevalent subclinical TB was over twice as common as symptomatic TB disease (48/2077, 2.3%, 95% CI 1.7–3.1% compared to 20/2077, 1.0%, 95% CI 0.6–1.5%). Subclinical TB prevalence was higher in people living with HIV (15/377, 4.0%, 95% CI 2.2–6.5%) compared to those who were HIV-negative (33/1696, 1.9%, 95% CI 1.3–2.7%; p=0.018). In regression analysis, HIV-positive status (377/2077, 18.2%) was associated with a two-fold increase in prevalent subclinical TB with 95% confidence intervals consistent with no association through to a four-fold increase (adjusted OR 2.00, 95% CI 0.99–4.01, p=0.052)., HIV-positive status was associated with and a five-fold increase in prevalent symptomatic TB (adjusted OR 5.05, 95% CI 2.22–11.59, p<0.001).  Conclusions: Most (70.6%) pulmonary TB diagnosed in household contacts in this setting was subclinical. HIV-positive status was likely associated with a two-fold increase in prevalent subclinical TB, and was associated with a five-fold increase in prevalent symptomatic TB. To improve early TB diagnosis in household contacts, more sensitive screening approaches may be needed, including chest radiography and universal sputum examination. |
| Introduction | | | |  |
| Background/rationale | 2 | Explain the scientific background and rationale for the investigation being reported | 5 | There is little data describing prevalence of subclinical TB in household contacts of people with TB, despite the known high prevalence of TB in this group.13 Estimates of the proportion of asymptomatic TB amongst all household contacts with TB vary from 50% to 96% across different settings.14.15,16 Over time, changing awareness of TB symptoms and improved case-detection for symptomatic patients may alter proportions of symptomatic and subclinical TB disease, necessitating an update of this estimate in the South African setting.  HIV is the mostan important risk factor for developing symptomatic TB disease,1,17 but the relationship between subclinical TB and HIV has not been fully explored. Typical TB symptoms are less common in people coinfected with HIV and TB.18,19,20 A better understanding of the relationship between subclinical TB and HIV is therefore important to inform screening strategies for people with and without HIV. |
| Objectives | 3 | State specific objectives, including any prespecified hypotheses | 5 | In this study, we determined the prevalence of subclinical pulmonary TB in household TB contacts in two South African provinces, and the association of subclinical pulmonary TB with HIV status. |
| Methods | | | |  |
| Study design | 4 | Present key elements of study design early in the paper | 1,6 | Prevalence of subclinical pulmonary tuberculosis and its association with HIV in household contacts of index tuberculosis patients in two South African provinces: a secondary, cross-sectional analysis of a cluster-randomised trial  This was a cross-sectional, secondary analysis of baseline data from a previously completed household cluster-randomised controlled trial (ISRCTN16006202), for which the protocol and results are published in full elsewhere.21,22 The trial compared two strategies for management of household contacts of people with microbiologically-confirmed TB: intensified home-based TB and HIV screening, and a referral letter strategy. The present study used the baseline data from the intervention arm of the trial only, where household contact participants were offered investigation for HIV and TB regardless of symptoms. |
| Setting | 5 | Describe the setting, locations, and relevant dates, including periods of recruitment, exposure, follow-up, and data collection | 6 | Data collection for the original trial took place between December 2016 and March 2019 in two South African provinces: Mangaung Municipality in Free State Province and Capricorn District in Limpopo Province. HIV prevalence was 17.034.4% and 10.919.3% in Mangaung Free State and Capricorn Limpopo provinces respectively on antenatal screening in 20179.23 TB incidence rates were 616 per 100,000 and 328 per 100,000 in Mangaung and Capricorn respectively in 2015 |
| Participants | 6 | (*a*) *Cohort study*—Give the eligibility criteria, and the sources and methods of selection of participants. Describe methods of follow-up  *Case-control study*—Give the eligibility criteria, and the sources and methods of case ascertainment and control selection. Give the rationale for the choice of cases and controls  *Cross-sectional study*—Give the eligibility criteria, and the sources and methods of selection of participants | NA  NA  6-7 | Index patients with TB were identified from government medical facilities, with those aged seven years or above required to have microbiologically-confirmed pulmonary TB for inclusion, while those under seven could have TB of any organany form of TB (microbiologically confirmed or clinically diagnosed) if diagnosed by a physician. Index patients could be living, or recently deceased, and were required to have been diagnosed with TB in the six weeks prior to recruitment. We excluded index patients who were incarcerated or in long-term inpatient care. Household contacts of index patients were defined as people living together within a set of rooms under a contiguous roof, linked by doorways and windows, through which air moved, and where household members had shared airspace by either sleeping overnight at least once, or had shared at least two meals in the same household as the index patient in the 14 days prior to the index patient’s diagnosis of TB.21,22 Those who did not meet this definition or who did not consent to participate were excluded. Household contacts under five were excluded from the present analysis as they were not required to produce sputum. There were no other exclusion criteria for household contacts or index cases. |
|  |  | (*b*) *Cohort study*—For matched studies, give matching criteria and number of exposed and unexposed  *Case-control study*—For matched studies, give matching criteria and the number of controls per case | NA  NA |  |
| Variables | 7 | Clearly define all outcomes, exposures, predictors, potential confounders, and effect modifiers. Give diagnostic criteria, if applicable | 7-9 | household contacts received: a questionnaire covering socio-demographic characteristics, risk factors for TB, presence of symptoms, self-reported diabetes, and history of previous TB and HIV treatment; point of care HIV testing, followed by measurement of CD4 count if positive; and those older than five were requested to produce a single sputum specimen regardless of their report of TB symptoms. This specimen was subjected to microbiological TB testing, including nucleic acid amplification test (NAAT with Xpert MTB/RIF or Xpert Ultra, Cepheid, Sunnyvale, USA), smear microscopy and mycobacterial growth indicator tube (MGIT) culture.  Microbiologically-confirmed pulmonary TB was defined as a household contact with a positive sputum auramine stained microscopy (scanty, or any + to +++ positive), or positive NAAT, or culture result speciated as M tuberculosis complex detected. TB symptoms were defined as the presence of any duration of cough, weight loss, night sweats, or fever.26 “Symptomatic pulmonary TB” was defined by microbiologically-confirmed TB in a participant who reported at least one of these four TB symptoms. “Subclinical pulmonary TB” was defined by microbiologically-confirmed pulmonary TB, in the absence of all four TB symptoms.  For multivariable models, a minimally-sufficient adjustment set of covariates consisting of household contact age (continuous), household contact sex, province of residence, and index case HIV status were identified through a directed acyclic graph (DAG) demonstrating putative pathways of association between the exposure of HIV status, and the outcome of subclinical TB (Figures 1a and 1b). |
| Data sources/ measurement | 8* | For each variable of interest, give sources of data and details of methods of assessment (measurement). Describe comparability of assessment methods if there is more than one group | 7 | Within 14 days of recruitment of index patients randomly allocated to the intensive screening arm, a home visit was undertaken where household contacts received: a questionnaire covering socio-demographic characteristics, risk factors for TB, presence of symptoms, self-reported diabetes, and history of previous TB and HIV treatment; point of care HIV testing, followed by measurement of CD4 count if positive; and those older than five were requested to produce a single sputum specimen regardless of their report of TB symptoms. This specimen was subjected to microbiological TB testing, including nucleic acid amplification test (NAAT with Xpert MTB/RIF or Xpert Ultra, Cepheid, Sunnyvale, USA), smear microscopy and mycobacterial growth indicator tube (MGIT) culture. All were asked to rinse their mouths with water prior to sputum collection. If the household contact was unable to produce a mucoid sputum specimen, we requested them to cough repeatedly and then spit whatever was in their mouth into the container and repeat this until ≥3ml was collected. The study laboratories were the public sector National Health Laboratory Service (NHLS) which has its own internal quality assurance programmes. At the time of the trial, South Africa was changing over Xpert MTB/RIF to Xpert MTB/RIF Ultra cartridges and the changeover differed at each site’s NHLS laboratory. |
| Bias | 9 | Describe any efforts to address potential sources of bias | 8-9 | For regression analysis, missing data was excluded listwise where missingness was less than 5%, with the assumption that data was missing at random.30 We planned that multiple imputation would be considered where an independent variable for regression analysis had missingness of 5% or more, though this was not required. To investigate the association between HIV status and TB disease state, univariable and multivariable binomial logistic regression models were constructed to estimate unadjusted and adjusted ORs and 95% CIs for two outcome comparisons: 1) subclinical TB versus no TB and 2) symptomatic TB versus no TB. To allow for clustering, province of residence was included as a fixed effect, and robust standard errors were used to account for household clustering.31,32 For multivariable models, a minimally-sufficient adjustment set of covariates consisting of household contact age (continuous), household contact sex, province of residence, and index case HIV status were identified through a directed acyclic graph (DAG) demonstrating putative pathways of association between the exposure of HIV status, and the outcome of subclinical TB (Figures 1a and 1b).24,33,34  A sensitivity analysis was conducted by excluding TB cases that were positive on smear only with no confirmation from other microbiological methods such as NAAT or culture. |
| Study size | 10 | Explain how the study size was arrived at | NA | Secondary analysis of trial data – sample size not applicable. |

Continued on next page

| Quantitative variables | 11 | Explain how quantitative variables were handled in the analyses. If applicable, describe which groupings were chosen and why | 8 | Data was analysed using R (version 4.1.2, R Core Development Team). We used descriptive statistics to summarise characteristics of household contacts, stratified by TB status. To assess for non-response bias, characteristics of household contacts included in the analysis were compared with those identified by index patients in the intervention arm at baseline, but who did not participate, and between contacts with and without microbiological TB results.  Prevalence of subclinical and symptomatic pulmonary TB were calculated as the number of cases in household contacts divided by the total number of household contacts for whom a microbiological TB result was available, with Clopper Pearson 95% confidence intervals (CIs).27,28 We also stratified prevalence estimates by province, HIV status and sex, and compared TB status using a two-proportions Z-test |
| --- | --- | --- | --- | --- |
| Statistical methods | 12 | (*a*) Describe all statistical methods, including those used to control for confounding | 8-9 | Data was analysed using R (version 4.1.2, R Core Development Team). We used descriptive statistics to summarise characteristics of household contacts, stratified by TB status. To assess for non-response bias, characteristics of household contacts included in the analysis were compared with those identified by index patients in the intervention arm at baseline, but who did not participate, and between contacts with and without microbiological TB results.  Prevalence of subclinical and symptomatic pulmonary TB were calculated as the number of cases in household contacts divided by the total number of household contacts for whom a microbiological TB result was available, with Clopper Pearson 95% confidence intervals (CIs).27,28 We also stratified prevalence estimates by province, HIV status and sex, and compared TB status using a two-proportions Z-test.29  For regression analysis, missing data was excluded listwise where missingness was less than 5%, with the assumption that data was missing at random.30 We planned that multiple imputation would be considered where an independent variable for regression analysis had missingness of 5% or more, though this was not required. To investigate the association between HIV status and TB disease state, univariable and multivariable binomial logistic regression models were constructed to estimate unadjusted and adjusted ORs and 95% CIs for two outcome comparisons: 1) subclinical TB versus no TB and 2) symptomatic TB versus no TB. To allow for clustering, province of residence was included as a fixed effect, and robust standard errors were used to account for household clustering.31,32 For multivariable models, a minimally-sufficient adjustment set of covariates consisting of household contact age (continuous), household contact sex, province of residence, and index case HIV status were identified through a directed acyclic graph (DAG) demonstrating putative pathways of association between the exposure of HIV status, and the outcome of subclinical TB (Figures 1a and 1b).24,33,34  A sensitivity analysis was conducted by excluding TB cases that were positive on smear only with no confirmation from other microbiological methods such as NAAT or culture. |
|  |  | (*b*) Describe any methods used to examine subgroups and interactions | NA | NA |
|  |  | (*c*) Explain how missing data were addressed | 8 | missing data was excluded listwise where missingness was less than 5%, with the assumption that data was missing at random.30 We planned that multiple imputation would be considered where an independent variable for regression analysis had missingness of 5% or more, though this was not required. |
|  |  | (*d*) *Cohort study*—If applicable, explain how loss to follow-up was addressed  *Case-control study*—If applicable, explain how matching of cases and controls was addressed  *Cross-sectional study*—If applicable, describe analytical methods taking account of sampling strategy | NA  NA  8 | To allow for clustering, province of residence was included as a fixed effect, and robust standard errors were used to account for household clustering |
|  |  | (*e*) Describe any sensitivity analyses | 9 | A sensitivity analysis was conducted by excluding TB cases that were positive on smear only with no confirmation from other microbiological methods such as NAAT or culture. |
| Results | | | | |
| Participants | 13* | (a) Report numbers of individuals at each stage of study—eg numbers potentially eligible, examined for eligibility, confirmed eligible, included in the study, completing follow-up, and analysed | 12-13 | A total of 4459 household contacts of 1032 index TB patients randomised to the intervention were identified, of whom 2993 (67.1%) household contacts from 923 households consented to participate. 445 household contacts under 5 years of age were excluded from these analyses as those under 5 were not required to produce sputum. Sputum was obtained for microbiological TB testing in 2146/2548 (84.2%) of the household contact participants aged ≥5 from which a result was available for at least one microbiological test (NAAT, smear or culture) for 2077 (81.5%) household contacts from 853 households (Figure 2). |
|  |  | (b) Give reasons for non-participation at each stage |  | Please see figure 2. |
|  |  | (c) Consider use of a flow diagram |  | Please see figure 2. |
| Descriptive data | 14* | (a) Give characteristics of study participants (eg demographic, clinical, social) and information on exposures and potential confounders | 14-15 | Table 1 provides characteristics of the 2077 household contact participants with a microbiological TB result, of whomich. 1190 (57.3%) were resident in Mangaung, and 887 (42.7%) in Capricorn. The median age of household contacts with a sputum result was 24 years (IQR 13 – 46), and 64.9% were female. 18.2% were HIV-positive, of whom 66.0% were taking antiretroviral therapy (ART). Missing values were common for the variables of CD4 count (80.4%), and ART status (23.9%) in household contacts living with HIV. There were low levels (<5%) of missing data for the remainder of the variables (Table 1). Characteristics of the 853 index patients are available in Appendix 1 (Additional file 1).  Please also see Table 1. |
|  |  | (b) Indicate number of participants with missing data for each variable of interest | 15 | Please see Table 1. |
|  |  | (c) *Cohort study*—Summarise follow-up time (eg, average and total amount) | NA |  |
| Outcome data | 15* | *Cohort study*—Report numbers of outcome events or summary measures over time | NA |  |
|  |  | *Case-control study—*Report numbers in each exposure category, or summary measures of exposure | NA |  |
|  |  | *Cross-sectional study—*Report numbers of outcome events or summary measures | 16 | Overall prevalence of pulmonary TB in household contacts was 68/2077 (3.3%, 95% CI 2.6–4.1%). Prevalence of subclinical pulmonary TB in household contacts was 48/2077 (2.3%, 95% CI 1.7–3.1%) and prevalence of symptomatic pulmonary TB was 20/2077 (1.0%, 95% CI 0.6–1.5%) - Figure 4. Subclinical pulmonary TB was 2.4 times as common as symptomatic pulmonary TB, with 70.6% (48/68) having subclinical TB (95% CI 58.3–81.0%).  Please also see Figure 4. |
| Main results | 16 | (*a*) Give unadjusted estimates and, if applicable, confounder-adjusted estimates and their precision (eg, 95% confidence interval). Make clear which confounders were adjusted for and why they were included | 18 | Please see Table 2. |
|  |  | (*b*) Report category boundaries when continuous variables were categorized | 15 | Please see table 1 |
|  |  | (*c*) If relevant, consider translating estimates of relative risk into absolute risk for a meaningful time period | NA |  |

Continued on next page

| Other analyses | 17 | Report other analyses done—eg analyses of subgroups and interactions, and sensitivity analyses | 18-19 | A sensitivity analysis was conducted by excluding TB cases that were positive on sputum smear only, with no confirmation from NAAT or culture. Nine TB cases were excluded on this basis, of which 2/9 were from people living with HIV. This resulted in an overall prevalence of pulmonary TB in household contacts of 59/2077 (2.8%, 95% CI 2.2–3.6%), a subclinical pulmonary TB prevalence of 39/2077 (1.9%, 95% CI 1.3–2.6%) and a symptomatic pulmonary TB prevalence remaining at 20/2077 (1.0%, 95% CI 0.6–1.5%). In sensitivity analysis, subclinical TB accounted for 66.1% (39/59) of TB cases amongst household contacts. HIV-positive status was associated with subclinical TB in adjusted analysis with an effect estimate of similar magnitude to that presented in the main results (adjusted OR 2.30, 95% CI 1.05 – 5.00, p=0.037) [Appendix 6]. |
| --- | --- | --- | --- | --- |
| Discussion | | | | |
| Key results | 18 | Summarise key results with reference to study objectives | 19-23 | Please see full text of pages 19-23 of the manuscripy (too long to copy-paste here) |
| Limitations | 19 | Discuss limitations of the study, taking into account sources of potential bias or imprecision. Discuss both direction and magnitude of any potential bias | 23-24 | The study also had several limitations. A high level of missingness for data on CD4 count and ART status in household contacts precluded further analysis of the relationship between immunosuppression and subclinical TB, which is an important area for future work, alongside exploration of additional potential risk factors for subclinical TB such as smoking status. Sputum screening was also incomplete. It is generally challenging to collect sputum samples from people in the community as some will struggle to produce a sample, especially children.48 The sputum collection rate achieved in our study was comparable to other studies in community and household settings.16,49 The younger median age of those with no sputum result could bias results in the direction of an underestimate of subclinical TB prevalence, given previous evidence of an association between younger age and subclinical TB.45,46 The changeover from Xpert MTB/RIF to Xpert MTB/RIF Ultra cartridges in South Africa part way through this study prevents further analysis of the relationship between the lowest 'trace' semiquantitative detection of the Ultra system and subclinical TB, however this should be explored in future work.  The p-value and 95% CI for the adjusted estimate of the association between HIV-positive status and subclinical TB marginally crossed the threshold typically assigned as statistically non-significant. We have avoided arbitrary dichotomisation of the findings using these thresholds, instead discussing the potential implications of the odds ratios and corresponding 95% CIs.50 The likelihood of a true association is further supported by results from a sensitivity analysis where TB cases that were diagnosed on smear alone were excluded in case of false positives (Appendix 6, Additional file 1). An effect estimate of similar magnitude was obtained for the association between HIV-positive status and subclinical TB, with upper and lower 95% confidence interval limits consistent with an association of 1- to 5-fold magnitude (adjusted OR 2.30, 95% CI 1.05 – 5.00, p=0.037). The generalisability of our findings outside of South Africa may be limited given the unique epidemiological context with significant dual burden of HIV and TB disease.9 In addition, the present study only looked at point-prevalent TB. There is potential for subclinical TB to be “unmasked” as symptomatic with symptom development over time, especially if duration of time spent in the subclinical phase of TB disease is short. Further, our ability to detect paucibacillary TB cases was limited by a lack of induced sputum or repeated sputum sampling. |
| Interpretation | 20 | Give a cautious overall interpretation of results considering objectives, limitations, multiplicity of analyses, results from similar studies, and other relevant evidence | 24 | We found that the prevalence of subclinical pulmonary TB in household contacts of index TB patients in two South African provinces substantially exceeded the prevalence of symptomatic pulmonary TB. HIV-positive status was likely associated with a two-fold increase in prevalent subclinical TB, and was associated with a five-fold increase in prevalent symptomatic TB. To improve early TB diagnosis in household contacts, more sensitive screening approaches may be needed such as, including chest radiography and universal sputum examination. Identifying and treating people living with subclinical TB should be a focus of future TB control efforts. This would ensure initiation of TB treatment earlier in the course of the disease, and is likely to reduce morbidity and mortality due to TB and importantly prevent TB transmission. |
| Generalisability | 21 | Discuss the generalisability (external validity) of the study results | 24 | The generalisability of our findings outside of South Africa may be limited given the unique epidemiological context with significant dual burden of HIV and TB disease |
| Other information | |  | | |
| Funding | 22 | Give the source of funding and the role of the funders for the present study and, if applicable, for the original study on which the present article is based | 26 | This research was supported by UK/South Africa Medical Research Council Newton Fund (006Newton TB) and Wellcome (200901/Z/16/Z to PM). NC was an In-Practice Fellow supported by the UK’s Department of Health and Social Care and the National Institute for Health Research (NIHR301000). The views expressed are those of the authors and not necessarily those of the NHS, the NIHR or the Department of Health and Social Care. |

*Give information separately for cases and controls in case-control studies and, if applicable, for exposed and unexposed groups in cohort and cross-sectional studies.
