# Supplementary material for: Prevalence of subclinical pulmonary tuberculosis and its association with HIV in household contacts of index tuberculosis patients in two South African provinces: a secondary, cross-sectional analysis of a cluster-randomised trial
Source: BMC Glob Public Health. 2023 Nov 1;1:21. doi: 10.1186/s44263-023-00022-5 (PMC11116238; doi:10.1186/s44263-023-00022-5)
Supplement: Supplementary file 2 — Additional file 2. Contains supplementary data and analyses in tables S1-6 as follows: Table S1. Demographic and clinical characteristics of index cases. Table S2. Comparison of household contact characteristics between those with and without a microbiological TB result. Table S3. Proportion of TB patients classified as subclinical based on individual interviewer symptom screens. Table S4. Association of HIV status with subclinical TB versus no TB assessed by multiple logistic regression (full model). Table S5. Association of HIV status with symptomatic TB versus no TB assessed by multiple logistic regression (full model). Table S6. Sensitivity analysis. [file 44263_2023_22_MOESM2_ESM.docx]

**Table S1:** **Demographic and clinical characteristics of index cases**

| Overall sample, N | | 853 |
| --- | --- | --- |
| Province (N, %) | Mangaung  Capricorn  Missing | 478 (56.0)  375 (44.0)  0 |
| Sex (N, %) | Male  Female  Missing | 522 (61.2)  331 (38.8)  0 |
| Age (years) [median, IQR]  Missing | | 37 (28 - 47)  0 |
| Age group | 0-4 years  5-9 years  10-15 years  16-24 years  25-34 years  35-44 years  45-54 years  55-64 years  65+ years  Missing | 12 (1.4)  3 (0.4)  9 (1.1)  111 (13.0)  221 (25.9)  245 (28.7)  118 (13.8)  84 (9.8)  50 (5.9)  0 |
| HIV status (N, %) | Positive  Negative  Missing | 461 (54.0)  353 (41.4)  39 (4.6) |
| Currently taking ART if HIV-positive (N, %) | Yes  No  Missing | 300 (65.1)  161 (34.9)  0 |

Percentages may not total 100 due to rounding (one decimal place). Age rounded to whole year. ART = anti-retroviral therapy. HIV = human immunodeficiency virus. IQR = interquartile range. N = number.

**Table S2:** **Comparison of household contact characteristics between those with and without a microbiological TB result**

|  | | **Has microbiological TB result** | |
| --- | --- | --- | --- |
|  |  | **Yes** | **No** |
| Overall sample (N, %) | | 2077 | 471 |
| Province (N, %) | Mangaung  Capricorn  Missing | 1190 (57.3)  887 (42.7)  0 | 252 (53.5)  219 (46.5)  0 |
| Sex (N, %) | Male  Female  Missing | 729 (35.1)  1348 (64.9)  0 | 204 (43.3)  267 (56.7)  0 |
| Age (years) [median, IQR]  Missing | | 24 (13-46)  0 | 10 (6-24)  0 |
| Age group (N,%) | 5-9 years  10-15 years  16-24 years  25-34 years  35-44 years  45-54 years  55-64 years  65+ years  Missing | 254 (12.2)  420 (20.2)  379 (18.2)  286 (13.8)  187 (9.0)  180 (8.7)  183 (8.8)  188 (9.1)  0 | 230 (48.8)  79 (16.8)  49 (10.4)  35 (7.4)  21 (4.5)  14 (3.0)  19 (4.0)  24 (5.1)  0 |
| Employment status | Currently employed  Not employed  Student  Other  Missing | 169 (8.1)  818 (39.4)  824 (39.7)  263 (12.7)  3 (0.1) | 19 (4.0)  106 (22.5)  246 (52.2)  100 (21.2)  0 |
| Average household size (mean, ±SD)  Missing | | 4.5 (2.6)  0 | 5.4 (2.7)  0 |
| HIV status (N,%) | Positive  Negative  Missing | 377 (18.2)  1696 (81.7)  4 (0.2) | 45 (9.6)  424 (0.0)  2 (0.4) |
| Currently taking ART if HIV-positive (N, %) | Yes  No  Missing | 249 (66.0)  38 (10.1)  90 (23.9) | 31 (68.8)  3 (6.7)  11 (24.4) |
| CD4 count if HIV-positive (N, %)  [cells/mm3] | <200  200-499  500+  Missing | 11 (2.9)  30 (8.0)  33 (8.8)  303 (80.4) | 0  5 (11.1)  2 (4.4)  38 (84.4) |
| Diabetes (N, %) | Yes  No  Missing | 39 (1.9)  2038 (98.1)  0 | 5 (1.1)  466 (98.9)  0 |
| Smoking status (N, %) | Currently smoke  Previously smoked  Never smoked  Missing | 228 (11.0)  39 (1.9)  1808 (87.0)  2 (0.1) | 16 (3.4)  6 (1.3)  448 (95.1)  1 (0.2) |
| Currently drinks alcohol (N, %) | Yes  No  Missing | 326 (15.7)  1749 (84.2)  2 (0.1) | 28 (5.9)  440 (93.4)  3 (0.6) |

Percentages may not total 100 due to rounding (one decimal place). Age rounded to whole year. ART = anti-retroviral therapy. HIV = human immunodeficiency virus. IQR = interquartile range. N = number. Restricted to age ≥5.

**Table S3:** **Proportion of TB patients classified as subclinical based on individual interviewer symptom screens**

| **Interviewer** | **Symptomatic TB patients** | **Subclinical TB patients** | **Proportion of TB patients that were subclinical (%)*** |
| --- | --- | --- | --- |
| 1 | 0 | 0 | NA |
| 2 | 4 | 4 | 50.0 |
| 3 | 0 | 1 | 100.0 |
| 4 | 0 | 0 | NA |
| 5 | 1 | 3 | 75.0 |
| 6 | 0 | 1 | 100.0 |
| 7 | 1 | 4 | 80.0 |
| 8 | 0 | 0 | NA |
| 9 | 1 | 2 | 66.7 |
| 10 | 0 | 0 | NA |
| 11 | 0 | 0 | NA |
| 12 | 0 | 0 | NA |
| 13 | 1 | 2 | 66.7 |
| 14 | 3 | 6 | 66.7 |
| 15 | 0 | 0 | NA |
| 16 | 0 | 0 | NA |
| 17 | 1 | 6 | 85.7 |
| 18 | 2 | 5 | 71.4 |
| 19 | 0 | 0 | NA |
| 20 | 0 | 0 | NA |
| 21 | 0 | 0 | NA |
| 22 | 6 | 14 | 70.0 |
| 23 | 0 | 0 | NA |
| 24 | 0 | 0 | NA |
| 25 | 0 | 0 | NA |
| 26 | 0 | 0 | NA |
| 27 | 0 | 0 | NA |

NA = not applicable. *Fisher’s exact test = p=0.98

**Table S4:** **Association of HIV status with subclinical TB versus no TB assessed by multiple logistic regression (full model)**

|  | | **Number/total sample^b^** | **Unadjusted OR (95% CI) [p-value]** | **Adjusted OR^c^ (95% CI)**  **[p-value]** |
| --- | --- | --- | --- | --- |
| Province | Mangaung  Capricorn | 28/1177  20/880 | Ref  0.95 (0.52 – 1.75) [0.879] | Ref  1.08 (0.57 – 2.05) [0.811] |
| Sex | Male  Female | 12/722  36/1335 | Ref  1.64 (0.84 – 3.19) [0.144] | Ref  1.43 (0.72 – 2.83) [0.302] |
| Age^a^ (years) | | N/A | 1.00 (0.99 – 1.01) [0.895] | 1.00 (0.98 – 1.01) [0.581] |
| **HIV status** | **Negative**  **Positive** | **33/1686**  **15/367** | **Ref**  **2.15** **(1.15 – 4.01) [0.016]** | **Ref**  **2.00 (0.99 – 4.01) [0.052]** |
| Index patient HIV status | Negative  Positive | 21/340  26/454 | Ref  0.99 (0.54 – 1.83) [0.985] | Ref  0.93 (0.49 – 1.76) [0.829] |

CI = confidence interval, N/A = not applicable, OR = odds ratio, Ref = reference category. Primary analysis highlighted in bold. Unadjusted odds ratios calculated using simple logistic regression via the glm() function in R, with province included as fixed term effect to adjust for clustering. Adjusted odds ratios calculated using multiple logistic regression via the glm() function in R. Robust clustered confidence intervals calculated based on clustering at household level, using the coeftest() function in R. ^a^Age included in the model as a continuous variable. ^b^Number and total sample number excludes missing data. Missing data dealt with listwise: ^c^1955/2057 included in the adjusted analysis.

**Table S5: Association of HIV status with symptomatic TB versus no TB assessed by multiple logistic regression (full model)**

|  | | **Number/total sample^b^** | **Unadjusted OR (95% CI) [p-value]** | **Adjusted OR^c^ (95% CI)**  **[p-value]** |
| --- | --- | --- | --- | --- |
| Province | Mangaung  Capricorn | 13/1162  7/867 | Ref  0.72 (0.29 – 1.80) [0.482] | Ref  0.82 (0.32 – 2.10) [0.677] |
| Sex | Male  Female | 7/717  13/1312 | Ref  1.02 (0.40 – 2.56) [0.968] | Ref  0.66 (0.28 – 1.60) [0.365] |
| Age^a^ (years) | | N/A | 1.02 (1.00 – 1.04) [0.014] | 1.02 (1.00 – 1.04) [0.052} |
| **HIV status** | **Negative**  **Positive** | **10/1663**  **10/362** | **Ref**  **4.62 (1.93 – 11.02) [<0.001]** | **Ref**  **5.05 (2.21 – 11.59) [<0.001]** |
| Index patient HIV status | Negative  Positive | 13/332  7/435 | Ref  0.41 (0.16 – 1.03) [0.058] | Ref  0.36 (0.15 – 0.89) [0.028] |

CI = confidence interval, N/A = not applicable, OR = odds ratio, Ref = reference category. Prespecified primary analysis highlighted in bold. Unadjusted odds ratios calculated using simple logistic regression via the glm() function in R, with province included as fixed term effect to adjust for clustering. Adjusted odds ratios calculated using multiple logistic regression via the glm() function in R. Robust clustered confidence intervals calculated based on clustering at household level, using the coeftest() function in R. ^a^Age included in the model as a continuous variable. ^b^Total sample number excludes missing data. Missing data dealt with listwise (complete case analysis): ^c^1929/2029 included in the adjusted analysis.

**Table S6: Sensitivity analysis**

**Association of HIV status with subclinical TB versus no TB assessed by multiple logistic regression (full model), excluding TB patients that were positive on smear alone**

|  | | **Number/total sample^b^** | **Adjusted OR^c^ (95% CI)**  **[p-value]** |
| --- | --- | --- | --- |
| Province | Mangaung  Capricorn | 23/1177  16/880 | Ref  1.06 (0.53 – 2.17) [0.864] |
| Sex | Male  Female | 10/722  29/1335 | Ref  1.49 (0.70 – 3.16) [0.301] |
| Age^a^ (years) | | N/A | 0.99 (0.97 – 1.00) [0.132] |
| **HIV status** | **Negative**  **Positive** | **26/1686**  **13/367** | **Ref**  **2.30 (1.05 – 5.00) [0.037]** |
| Index patient HIV status | Negative  Positive | 16/332  22/435 | Ref  0.97 (0.48 – 1.97) [0.932] |

CI = confidence interval, N/A = not applicable, OR = odds ratio, Ref = reference category. Primary analysis highlighted in bold. Adjusted odds ratios calculated using multiple logistic regression via the glm() function in R. Robust clustered confidence intervals calculated based on clustering at household level, using the coeftest() function in R. ^a^Age included in the model as a continuous variable. ^b^Number and total sample number excludes missing data. Missing data dealt with listwise: ^c^1955/2057 included in the adjusted analysis.
